# Creative social prescribing groups during lockdown: a photo-elicitation evaluation of group users' experiences

**DOI:** 10.3389/fpubh.2025.1702170

**Published:** 2026-01-27

**Authors:** Lindsey Bishop-Edwards, Elizabeth Taylor Buck, Jane Stockdale, Scott Weich

**Affiliations:** Division of Population Health, Sheffield Centre for Health and Related Research, School of Medicine and Population Health, University of Sheffield, Sheffield, United Kingdom

**Keywords:** connection, COVID, hybrid, lockdown, mental health, social prescribing

## Abstract

Previous research has examined the experiences of voluntary, community, and social enterprise (VCSE) group providers, but the voices of group users, especially those recovering from, or living with SMI, are sparse. This study addresses that gap, using the COVID-19 pandemic lockdown as a natural experiment to gain insights into the experiences of individuals participating in VCSE-run creative and social groups, and comparing this with the experiences of providers. We conducted photo-elicitation focus groups to explore group members' perspectives. Lockdown highlighted to group members what they valued most, and therefore what they missed about attending groups. Participants reflected on how significant their group was to their experiences of social connectedness and belonging. Group members' experiences differed significantly from providers' experiences of delivering online groups during lockdown. In some cases lockdown exacerbated existing challenges for marginalized individuals, highlighting the critical importance of compassionate, skilled, and well-informed group leadership. The needs of marginalized populations and the risk that these may be overshadowed by other public health priorities were highlighted. Our findings underline the urgency of developing inclusive policies and practices that prioritize voices of marginalized groups. By listening to these voices, policymakers and group leaders can create fairer and more responsive support systems.

## Introduction

During the pandemic, community groups run by Voluntary Community and Social Enterprise (VCSE) organizations were forced to close or alter the provision they offered, reducing contact for group members and contributing to the profound sense of isolation some people experienced during lockdown. This may have been particularly challenging for people with mental health difficulties, who rely on community groups not only for connection and purpose, but also for the “creative identity” that connecting with others can provide (1). A survey of VSCE providers operating in South West Yorkshire found that the isolation of lockdown helped providers to see that they may have underestimated or taken for granted the importance of the interpersonal connection their groups offered (13).

Lockdown created the conditions for a natural experiment to understand more about the mechanisms through which creative community groups improve the mental health of group users who have experienced Serious Mental Illness (SMI). To do this, and to find out more about the impact of changes in group delivery during lockdown, we conducted focus groups with people who had been engaged with VCSE groups before, during and after the imposition of social restrictions. All participants were recruited from community-based groups supported by Creative Minds, a charity embedded within South West Yorkshire Partnership NHS Foundation Trust (SWYPFT). Creative Minds co-funds and supports community activities delivered by partners in the South West Yorkshire region that enhance regular provision. The activities are accessible and appropriate for people with severe mental health needs, learning disabilities, and neurodiversity, providing opportunities for engagement as equals for mutual benefit (2). The range of creative group activities covered by CM funding is vast and includes arts, sports, community and social gatherings, theater, youth work and gardening, and these are typically regular in-person groups that meet one or more times a week. During lockdown however, the groups were forced to change their offer and implement things like zoom meetings, postal art packs, at home crafting, gardening activities, online games and letter writing.

Using photo-elicitation, we sought to capture group user perspectives on the change to the provision of their creative group and the impact this had on them and their mental health recovery process. The changes that were imposed as a consequence of lockdown provided a unique opportunity for group members to reflect on what they missed, on what they gain from the groups they attend and how they feel now, in the post-lockdown era. This insight may be particularly important given a significant move to remote mental health service delivery post-lockdown (3) and in light of suggestions that hybrid delivery may work better than online provision due to low engagement with online tools (4). Comparing these data with previous findings about provider experiences of changing service provision in lockdown allowed for further insight into group members' lived experiences.

## Materials and methods

### Design

Focus groups were held with members of groups who had received support or funding from CM. Photo-elicitation is a qualitative method that involves participants looking at, choosing, or taking photographs to help them explore and express their own experiences. Photo-elicitation in health research is appropriate and effective when aiming to promote discussion and reflection about emotions, memories and ideas (5). Moreover, it allows for greater participant agency (6). This method was used to prompt memory and to explore what people felt they lost, or gained, when the community groups they were part of changed or closed during lockdown. There was also a focus on the impact that the changes had on their mental health and recovery journey. Group leaders were not present during the focus groups, ensuring that participants could speak freely and were not under pressure to “please” the group leaders.

Images used for the photo-elicitation included: photographs of activity packs sent out by VCSE group providers; images of output that were produced by some group members in lockdown; images of lockdown such as empty streets, locked doors, people talking through windows, and being home alone. These images were sourced from published news articles and features that were still available online. The images were designed to cover a broad range of issues that may have been relevant to participants and were conceptualized in categories such as the shutting down of the external world, isolation in the home, mask wearing and medical risk, use of technology, home schooling, at home activities, mental health and positive images. All images were laid out at the start of focus groups and participants were given time to look at the images. All participants had access to all images to ensure consistency between groups. They were encouraged to pick out whichever images resonated with them, for any reason. The images they chose were what they first spoke about and reflected on.

To enhance the agency of the participants, we used an “auto-driving” technique. This technique involves having no prior topic guide; instead the participants “drove” the discussions organically by choosing which pictures to discuss and in what order. This technique allows freer thought and displaces some of the researcher-participant power differential (7). Two facilitators ran each focus group. This ensured enough resource to make notes, guide where needed and support anyone who may wish to leave or take a break. Researchers listened to the stories of the participants, asked clarifying questions and reminded participants of the aim of the discussion, if necessary. Focus group discussion lasted between 40 and 60 min.

### Lived experience contribution

Significant parts of the design were taken to a mental health lived experience group, the Lived Experience Advisory Panel (LEAP) for their advice and feedback. They commented on the images chosen and some of these were changed or replaced as a result of the feedback. Their feedback ensured that there was a broad coverage of themes represented by the images and that the images conveyed what they were intended to convey. For example, an image of a lonely person was interpreted as such. Additionally, the LEAP consulted on the running of the focus groups, the content and accommodations that needed to be included. Some examples were being mindful of participants feeling safe and having space to take a break, being mindful of asking participants to speak about a potentially uncomfortable time in their lives, using appropriate language, expressing gratitude, appropriate ice breakers and being very clear about confidentiality and creating a safe space for discussion. All feedback and advice was utilized.

### Participants

All active CM groups were contacted via email through CM, and then acted as gatekeepers to their groups, contacting the researcher directly if they thought people attending their group might be appropriate and interested in the research. Group leaders spoke to their groups about the research and invited the researchers to visit the group and tell them about the focus groups. If the group's members were happy to go ahead, a date for the research was set and those who chose to participate attended the focus groups. The recruitment target was between 4 and 8 focus groups with no more than 8 people in a focus group. Groups with venues suitable for focus groups to be held were prioritized to enable a safe space for these focus groups to be conducted. We responded to and visited all groups who contacted us.

Participants were required to be an established member of a group that had been supported by CM prior to the pandemic lockdown. A formal mental health diagnosis was not specified as an inclusion criterion, however the groups sampled were inclusive and some were specifically designed for people with mental health issues, learning disabilities (LD), neurodiversity or a mix of conditions. No specific diagnostic or demographic data was asked of participants. This was strongly advised by group leaders and the LEAP advisory group as invasive questionnaires were thought to actively deter group members from taking part. We undertook six focus groups across four organizations. Twenty-two participants took part in the focus groups. All participants attended in-person focus groups. One participant who was not able to attend on the day due to personal reasons requested to submit feedback via email. Additional ethics approval was sought for this and the person's group leader forwarded the feedback to the researcher. This was coded alongside the focus group data. All participants received a shopping voucher as a thank you for their time.

## Ethical approvals

Ethical approval was sought and granted by The University of Sheffield, reference Number 049790.

### Analysis

Data from focus groups was recorded, and transcribed verbatim. The additional written feedback were analyzed thematically using inductive thematic Analysis (8). Full analysis was done by two researchers (LBE and JS) using the transcripts, analytic software was not used due to the sample size. Initial coding was undertaken jointly between the researchers after individual familiarization and coding. Coding and notes were shared for transparency and similarities and differences in interpretation and context were challenged and discussed between the researchers and adapted as needed to formulate the coding framework. A third researcher (ETB) then joined a critical discussion with the coding researchers in which the codes and key themes were developed and refined. Themes are first outlined below and then compared to previous research exploring the providers' views on the impact of lockdown on their provision and members (13). This comparison aimed to add depth to the understanding of the experiences of group users.

## Results

Social connectedness and belonging as part of a group were predominant themes that ran throughout the data. Participants were also able to reflect on the factors that they believed were important contributors to the success and cohesion of the group, including the creative activity being undertaken, the wider opportunities and connections available via the group, and the integral role of the group leader.

### Keeping connection alive

The primary positive experience reported by participants about going online in lockdown was the continuation of some semblance of connection and belonging to their group and a relief that there was not a total cutting off from their “gang”.

*I mean I was just grateful we did get to do online* (FG4)

Participants recalled some of the adaptations that had been made in lockdown by their group leaders, for example, making sure there was an alternative provision online, listening to shared music and having online “coffee” breaks. This was seen as providing something akin to the connection they felt when in the in-person group.

…*you felt linked…we were isolated, so you were linked to people that you knew and there was a little bit of banter*. (FG1)

Participants reported varying levels of satisfaction with online activities, but regardless of this it was clear that the element they most valued was the feelings of camaraderie. Some commented that, despite enjoying the online version, they still thought that the group was “better face to face” (FG3) as it was more collective.

When reflecting on the easing of lockdown and the return to number restricted in-person groups, some participants reported that they loved being back together but missed the feeling of being in the whole group.

*It was a bit, still a bit weird…that we weren't like all together all the time*. (FG3)

Smaller returning numbers were also the case for some groups due to some members not feeling able to return due to worries about health and infection, loss of confidence, loss of family and age.

*But just after a certain point it just starts to feel more, more and more awkward and strange to come back*. (FG5)

*Yeah because covid's over, they've just expected to go back…that's what I mean, for the people that's actually lost people, it's a lot more harder for them really isn't it?* (FG5)

Some participants also expressed a feeling that the groups “togetherness” was not quite the same without their original members and that new members tended to be more transient. There was a sense of missing those people and of missing some of the solid, reliable connections they used to have.

*They come but they don't sort of have the staying power as the original group… a lot flit in, flit out, yeah*. (FG1)

These examples show how important the in-person experience was to group members' feelings of connection and how having that established connection allowed it to continue online, providing some continuity for members. The connections formed pre-lockdown were described as “*something that was always gonna be there*” (FG5), showing how vital the groups in question had been in providing opportunities to form such bonds.

#### Reflections on connection

Participants valued the connection and companionship of the group. In some cases participants talked about how this led them to think more about the nature of connection where they may not have otherwise thought about it.

*it kind of I think started to make me realize yeah what human contact actually means kind of thing?* (FG4)

Lockdown also led some participants to reflect on connection within society and to appreciate the additional communication and care that had come about in lockdown.

*[Y]ou saw the old spirit come back where you'd help your neighbours yourself, you know* (FG5).

The lens of lockdown allowed for these additional reflections on what it means to be human, the value of human connection and the importance of their creative groups. As previously described, even those who enjoyed elements of online groups and lockdown, eventually wanted to reconnect in person. This suggests that the creative groups are offering benefits, connection and experiences that are not available elsewhere in the lives of those who attend, meaning that to regain that membership was “*a happy day in history*” (FG4).

### Sense of belonging

There was in some groups a degree of debate or uncertainty about the types of activities they were sent out and how people had stayed in touch. However, in contrast, there were much clearer recollections and certainty about the feeling of belonging and the desire to get back to the environment where members felt they belonged. Belonging therefore became a theme, both in discussions about the strong connection participants felt in attending community settings, and through reflection on what was lost during COVID-19 restrictions.

*I want the real thing, real feeling… It [being online] doesn't feel uncomfortable, I could do it, but it's just I just like to do it personally…* (FG4)

…*when you've been used to coming into a group for years to work with people that you've really associated with, it were like you'd had your arms or legs cut off to be honest, but seeing other people's faces, it makes a difference doesn't it* (FG5)

The importance of their respective groups to the participants was clear. There were various factors that contributed to the feelings of belonging and positive effects of the in-person groups. These included feeling seen and known within the group; a place to be themselves, a place of friendship, where people were supportive and accepting of differences and individual needs.

One participant described feeling judged by a lot of people in their life, but that the group they attended welcomed and accepted them for who they were:

…*we all know what I'm like and you just accept it …I feel so nervous…round everybody I really do and … you all don't make me feel like that* (FG5)

Whilst another participant describes the effect of attending the group on their mood:

*I can come here feeling a bit crap…yeah, it really does help…I think it's comforting* (FG5)

Other participants talked about group members as being their friends and missing them when they could not go to their regular groups, showing a strong social belonging that the creative groups can provide opportunity for:

*I did miss it, I always miss[ed] my friends*. (FG4)

*You missed many friends, you missed because the weekly session was shut down*. (FG6)

*It's my, you know, gang. (FG4)*

Care, understanding of their fellow group members, altruism and empathy were also clearly demonstrated. Group members looked out for each other, considered each other's lives and provided peer support to one another. They described taking note of how other people looked in online groups as a way of checking others' welfare, and prioritizing the return of those who they thought needed in-person groups the most, despite wanting to return themselves.

…*people there were looking well…we all looked well during it didn't we* (FG1)

*I didn't even try to come in until things were much more open because…I knew that it would be, honestly it would be wrong for me to try to come in; to take up the spot that for somebody who genuinely needed it more, like a lot more than I did*. (FG5)

These types of decisions arose as lockdown was starting to be lifted but there were restrictions on the amount of people who could gather together in one space. Some group members took it upon themselves to decide who was most in need of returning to the group, showing empathy, care and peer support.

### Magnification of marginalization

National lockdown helped participants reflect on the extent to which they had had to live with restrictions even before the pandemic and how little thought was given to their needs during the national crisis. Participants reflected that the restrictions of lockdown did not feel entirely new to them due to the challenges they faced in their lives before the pandemic, and they felt that when lockdown came, the rest of the world found out what life was like for them. This seemed to be integrally connected to their experiences of “normal,” non-COVID life which was, in some groups, reported to include isolation, stigma, lack of support and restriction. This was linked to a range of reasons including age and health, transport, neurodiversity, mental health and learning disability, or in other words, what is needed to meet their specific needs.

*…there were no, no really any support so I just basically really went downhill from there. (FG5)*

*I don't think I had a lockdown. I just sat at home on my computer. (FG4)*

Participants reported that once restrictions such as limiting interpersonal contact and wearing masks were mandated, they found that they were perhaps impacted more than other members of society, for example due to existing anxieties or limited social networks and isolation

*Even after lockdown the doctors still insisting on masks … it set off all my anxiety (FG3)*

*The older ones, I think they just got, lost the will to sort of get out and about…. They've broken the link. (FG1)*

Frustration was expressed that they had been forced to stay apart when some saw no good reason to do this. Some felt that, as an isolated group of people, who needed the contact of the group, they should have been allowed to continue.

…*for like groups like ours that aren't like massively big they should've had more clearer exemptions and because a lot of us we live on our own don't we so, we wouldn't have been seeing anyone else anyway (FG3)*

The lens that lockdown imposed clearly highlighted how the blanket rules applied during this period lacked understanding, consideration and inclusion of the needs of these groups of people, leading to a perpetuation of marginalization.

### The role of leadership

All groups spoke positively about the leadership of their group including the periods before, during and after lockdown and this was linked to the success of the group. Certain qualities, roles and styles of leadership were noted as helpful for a successful and sustainable group. In particular, leaders were valued for exhibiting warmth, helping group connection and cohesion, selecting appropriate activities, and understanding individual likes and dislikes.

## Interpersonal skill

The type of person that led the group, how they led the group and their interaction with the group members was described by numerous participants. In particular participants appreciated warmth and good interpersonal skills. This was inevitably linked to the feel of the group and whether people felt cared about and comfortable in that environment. This was the case before, during and after lockdown.

*[H]e used to talk to people a lot and find out how they were…He's got a very warm character hasn't he (FG1)*

Knowing people as individuals and communicating appropriately with them was seen as important and helped with any potentially difficult interactions

*[W]e had this meeting about the funding, well, paying our dues and demands but sort of, I don't know he's got a lovely way with him … how to get things across to you. (FG2)*

## Group opportunities and individualization

Group members reflected on and spoke about their group leaders with high regard and appreciation for them as a person, but also for the range of roles and opportunities that they provide for their group members. This included a wide variety of references. Reflecting on before lockdown, groups talked about leaders considering the preferences of the group, facilitating social engagement out in the community, having a busy social environment at the group and setting up buddy systems for new members.

*Just when you're new they attach you to someone to guide you and show you how to do it. (FG6)*

Reflecting on both before and after lockdown, group members talked about the links, contacts and innovation of their group leaders. Participants highlighted that varied and skilled input from the group leader made a significant difference to the quality of their experience and opportunities within the group. This included promotion through local media, community arts initiatives, in group games that supported social connection and friendship, showcases and trips out and about.

…*we wrote down a memory anonymously, we had to guess who might have said that memory (FG4)*

…*we're so lucky (FG5)*

## Appropriate activities

During lockdown, group members recalled the types of activities that their group leader sent out:

*…arts and crafts kind of thing yeah which we normally do, so yeah we do a bit of that…all between us we wrote on or drew something yeah or played little games… something about hobbies and things or things that relate to us (FG4)*

Activities were often described that had elements of connection and sharing, showing the aim of the group leader during lockdown went beyond just sending out things “to do,” but also sending things that would “connect.” Participants also mentioned leaders doing 1:1 calls and visits, sending activity packs and other ideas for things to do, initiating check ins, and moving online for live and catch-up sessions. These examples reflect the multiple focus that is required from leaders of successful groups, and the need to be able to embody numerous roles such as link to the community, peer support, innovator and provider.

## Value of group activities

The suspension of in-person groups prompted reflection on how much participants appreciated their groups and what it was that made it valuable to them. This included the activity itself, for example enjoying being immersed in music, or gaining physical and mental benefits from being outdoors, or having the opportunity to engage in creative activities and explore their creative side.

*[it's] very peaceful. An oasis really (FG6)*

*I am creative, I've got good stories and stuff (FG4)*

The opportunities to participate in creative activity and application, both in and out of lockdown, is significant to the experience of the group members, and can be seen to contribute to a sense of accomplishment and identity (9).

### Comparison of group providers and group members

Drawing on the findings from a study looking at VCSE group providers views on changing their provision in lockdown (13), a comparison was undertaken. Table 1 shows the key factors of lockdown and the extent to which the experiences of group providers and group members were convergent, partially convergent, or not convergent at all.

**Table 1:** Key factors of lockdown and level of convergence between group providers and group members.

**Factors of lockdown**	**Group providers**	**Group members**	**Relationship**
Initial response to lockdown	Lots to do including acquiring new technology skills and knowledge. A sense of being very busy	Less to do, creating a sense of loss and emptiness	Non-convergent
	Needed to adapt to new group formats	Needed to adapt to new group formats	Convergent
Impact of lockdown rules	Required to monitor and follow ever changing rules guidelines. A practical challenge	Required to follow ever changing rules and felt marginalized by those rules. An emotional and practical challenge	Partially convergent
	Experienced less autonomy in group formats and activities	Experienced less autonomy in doing things that helped themselves	Convergent
The needs of the group	Experienced stress due to different people needing different things, whilst also feeling less able to assess how people were due to less direct feedback	Experienced online sessions as a good opportunity to see how others were doing and felt confident in this assessment	Non-convergent
Connection	Realized connection was important to group members	Confirmed that connection was at the core of the group membership	Convergent
	Focused on the emotional labor: Active monitoring of group members and a care role. Amounted to significant extra responsibility	Focused on the emotional impact: Loss of what had previously been offered and emotional gratitude for what was being offered	Non-convergent
Activities provided	Did their best to innovate and find group appropriate creative tasks that had elements of connection	Appreciated the activities and the glimpses of connection they provided	Partially convergent
Role of the leaders	Numerous existing roles plus new ones such as safeguarding, welfare checks, online and at home activities	Appreciated the many roles and skills of the leaders and admired their calm and containing manner	Non-convergent

▭ non-convergent; ▭ convergent; ▭ partially convergent.

#### Convergent findings

The analysis showed that both group providers and group members felt they had to adapt to the new group formats, and as such both parties felt a loss of autonomy. Also, the importance of connection was highlighted or reaffirmed for both groups.

#### Partially convergent

Some findings within the analysis of group user date appeared to be partially convergent with the provider's findings. For example, the providers sought to find activities that would allow connection between members, and the members saw and appreciated this. Also, both groups were required to follow the lockdown rules, however, for the providers this was primarily a practical challenge whereas for members there was an emotional impact as they felt further marginalized and unseen.

#### Non-convergent findings

The analysis showed the ways in which the experiences and perspectives of group providers and group members differed significantly. While providers were focused on the emotional labor of adapting and maintaining groups, group members were more focussed on the emotional loss of no longer meeting face to face. Additionally, group leaders found it harder to assess how their adapted activities were landing whereas group members found the online forums gave them a sense of being able to check in with each other. Finally, on an intrapersonal level, the providers felt themselves to be scrambling frantically to pull together safe and appropriate lockdown activities. In contrast, on an interpersonal level, the group members experienced them as calm and containing.

## Discussion

Our research found that the lockdown conditions exacerbated existing challenges faced by marginalized individuals, echoing previous findings that pandemic restrictions disproportionately impacted those already experiencing social and economic inequalities (10). However, this study also highlights the vital role of VCSE-run groups in mitigating some of these challenges. Despite restrictions on in-person contact, these groups offered members a profound sense of belongingness, connection, routine and friendship. This may be seen as community groups delivering a means of building community resilience which is considered a protective factor in times of public health emergency (11). The lens of the pandemic brought renewed clarity to the meaning and value of these groups, particularly the interpersonal relationships and peer support that often extended beyond the formal boundaries of group activity.

The findings also underscore the central importance of connectedness and demonstrate that creating connection requires thoughtful, context-specific delivery aligned with the diverse needs of group members. This has significant implications for future public health strategies, especially during emergencies such as pandemics, where one-size-fits-all strategies may fail to engage or support vulnerable or marginalized communities.

Future research should prioritize understanding the longitudinal impact of such interventions from the perspective of participants themselves. Given the growing financial pressures on VCSE organizations, it is also vital to evidence how these services support individuals and to capture the nuanced ways in which they contribute to connection and recovery, making this a key area for ongoing investigation.

It is appropriate to acknowledge a limitation of this study, which is that we only spoke to people who were established members of a VCSE group, in one geographical area. As such, the perspectives of individuals who previously attended but did not continue, those who chose not to engage with the group, or those unaware of or unable to access such services is not represented. Future research should aim to include these underrepresented voices to develop a more inclusive understanding of access and engagement with VCSE provision. It would be helpful to replicate the research in other geographical areas to support the value of community groups beyond the South Yorkshire area, nationally and internationally.

In addition to highlighting the value of VCSE groups, our study also sheds light on what makes them effective. Participant reflections across pre-lockdown, lockdown, and post-lockdown periods revealed that consistency, continuity, and group longevity were especially important. This is really important for group members in general, and is particularly so during times of heightened isolation, whether during a lockdown, through ill health, physical isolation or a mental health relapse.

Comparisons with previous research (13) show that group sustainability is viewed differently depending on roles: for group leaders, it hinges on funding and resources whereas for group members it is grounded in inclusion, support, skilled leadership and peer bonds.

Activities delivered within the groups seemed to have purposeful elements that were included to engage people as individuals and to connect people as a group. The value placed on group leaders by group members was striking: they were not only facilitators, but also organizers, fundraisers, and crucially, people who understood and responded to group members' specific needs.

Lastly, the appreciation expressed for the provision that was laid on during lockdown underscores the importance of flexible, hybrid models of community engagement. While in-person interaction may be the preference for many, online formats can provided a valuable alternative, particularly for those unable to attend physically. This has implications for how VCSE services might continue to offer inclusive, adaptive support in the face of future disruptions, and for people who for other reasons cannot attend groups in person.

## Conclusion

This research found that VSCE group members felt meaningfully supported during lockdown by intuitive, compassionate connection, despite leaders having being previously less aware of its importance. For people with learning disabilities, neurodiversity, or mental health issues, the experience of lockdown often echoed pre-existing challenges, reinforcing the sense that pandemic restrictions merely intensified ongoing marginalization. Public health decisions during the pandemic, such as mandatory mask-wearing, and policies that increased social isolation, frequently failed to account for the needs of some already marginalized people. This oversight may have contributed to concerning trends observed during the pandemic, such as increased suicide rates and higher rates of involuntary hospital admissions (12).

While VSCE leaders providing community-based groups strove to adapt and sustain community-based group activities during lockdown, and their efforts were appreciated, they were generally seen as a poor substitution for in-person connections. Before, during and after lockdown, participants valued leaders who exhibited warmth, teaching and negotiation skills, and a good understanding of the unique circumstances of the individual group members. Most importantly, group members emphasized the significance of the interpersonal relationships, connection, and strong sense of belonging that they gained from being a member of their creative VSCE group.

## Data Availability

The datasets presented in this article are not readily available because the data is difficult to anonymize due to the size and localities of the community groups. Confidentiality is vital to future engagement with community groups. Requests to access the datasets should be directed to l.j.bishop-edwards@sheffield.ac.uk.

## References

[1] Baxter L Burton A Fancourt D. Community and cultural engagement for people with lived experience of mental health conditions: what are the barriers and enablers? BMC Psychol. (2022) 10:71. doi: 10.1186/s40359-022-00775-y 35296361 PMC8928686

[2] Walters P. Creative minds: developing supportive creative opportunities in our communities. Mental Health Soc Inclus. (2015) 19:30–7. doi: 10.1108/MHSI-12-2014-0041

[3] Oliver A Chandler E Gillard J. Impact of digital inclusion initiative to facilitate access to mental health services: service user interview study. JMIR Mental Health. (2024) 11:e51315. doi: 10.2196/51315 39058547 PMC11316150

[4] Chen K Huang JJ Torous J. Hybrid care in mental health: a framework for understanding care, research, and future opportunities. NPP—Digit Psychiatry Neurosci. (2024) 2:16. doi: 10.1038/s44277-024-00016-7 41360926 PMC12624923

[5] Glaw X Inder K Kable A Hazelton M. Visual methodologies in qualitative research: autophotography and photo elicitation applied to mental health research. Int J Qualit Methods. (2017) 16:1–8. doi: 10.1177/1609406917748215

[6] Fawns T. The photo-elicitation interview as a multimodal site for reflexivity. In:Reavey F, editor. A Handbook of Visual Methods in Psychology: Using and Interpreting Images in Qualitative Research. London: Routledge (2020). p. 487–501. doi: 10.4324/9781351032063-3328

[7] Ford K Bray L Water T Dickinson A Arnott J. Auto-driven photo elicitation interviews in research with children: ethical and practical considerations. Comprehens Child Adolesc Nurs. (2017) 40:111–25. doi: 10.1080/24694193.2016.1273977 29318953

[8] Braun V Clarke V. Thematic analysis. In:Cooper H Camic PM Long DL Panter AT Rindskopf D Sher KJ, editors. APA Handbook of Research Methods in Psychology, Vol. 2. Research Designs: Quantitative, Qualitative, Neuropsychological, and Biological. Washington, DC: American Psychological Association (2012). p. 57–71.

[9] Goodman Casanova JM Guzman-Parra J Mayoral F Cuesta-Lozano D. Community-based art groups in mental health recovery: a systematic review and narrative synthesis. J Psychiatr Ment Health Nurs. (2023) 31:158–73. doi: 10.1111/jpm.12970 37638556

[10] Scott S McGowan VJ Wildman JM Bidmead E Hartly J Mathews C . COVID-19 and the role of voluntary, community, and social enterprises in northern England in responding to the needs of marginalised communities: a qualitative focus group study. Lancet. (2022) 400:S78. doi: 10.1016/S0140-6736(22)02288-7 36930026 PMC9691055

[11] Hall CE Wehling H Stansfield J South J Brooks SK Greenberg N . Examining the role of community resilience and social capital on mental health in public health emergency and disaster response: a scoping review. BMC Public Health. (2023) 23:2482. doi: 10.1186/s12889-023-17242-x 38082247 PMC10714503

[12] Bonello F Zammit D Grech A Camilleri V Cremona R. Effect of COVID-19 pandemic on mental health hospital admissions: comparative population-based study. BJPsych Open. (2021) 7:e141. doi: 10.1192/bjo.2021.975 34340723 PMC8365103

[13] Bishop-Edwards L Taylor Buck E Weich S. The delivery of creative socially prescribed activities for people with serious mental health needs during lockdown: Learning about remote, digital and hybrid delivery. PLoS ONE. (2024) 19:e0301550. doi: 10.1371/journal.pone.0301550 38748673 PMC11095676

